# Current evidence on the burden of head and neck cancers in Nigeria

**DOI:** 10.1186/1758-3284-1-14

**Published:** 2009-05-28

**Authors:** Opubo B da Lilly-Tariah, Abayomi O Somefun, Wasiu L Adeyemo

**Affiliations:** 1 Department of Surgery, College of Health Sciences, University of Port Harcourt, Port Harcourt, Nigeria; 2 Department of Surgery, College of Medicine, University of Lagos, Lagos, Nigeria; 3 Department of Oral and Maxillofacial Surgery, College of Medicine, University of Lagos, Lagos, Nigeria

## Abstract

**Background:**

Head and neck cancers (HNC) constitute 5–8% of total body cancers in Europe and America. It is difficult to appreciate the problem of cancers in Nigeria because most studies available are hospital-based studies. The aim of this study is to highlight current evidence on the burden of head and neck cancers in Nigeria based on literature review and to discuss potential health care actions to improve management.

**Methods:**

A literature search using Medline was conducted for publications on head and neck cancer in Nigeria. Identified publications were manually searched for additional relevant non-Medline articles or abstracts. The full-texts of these articles were thoroughly examined for the occurrence, distribution, identified risks factors, presentations, diagnostic method, treatment, prognosis and challenges associated with the management of HNC.

**Results:**

A total of twenty-seven relevant published articles on Head and neck cancers from 1968 to 2008 were reviewed. The age of patients with HNC ranged from nine months to over 80 years with peak between 3–6^th ^decade of life. The male to female ratio ranged from 1:1 to 2.3:1. Identified risks factors were scanty, namely kola nuts and tobacco chewing, tobacco smoking, farming, viral infections, alcohol and smoking. Reports on the overall pattern of Head and neck cancers from different regions of the country cited nasopharynx as the commonest site for HNC, the sino-nasal is the second commonest while larynx, is the third commonly affected site. The majority of HNC was epithelial in origin and was mostly squamous cell carcinoma. Late presentation with advanced disease is common and treatment in most cases is palliative either with surgery or chemotherapy, and radiotherapy when available. There are few reports on the outcome of HNC treatment in Nigeria.

**Conclusion:**

The burden of managing HNC in Nigeria is enormous and the government should set up the National Cancer Institute with a view of educating the public on cancer prevention, detection and treatment.

## Introduction

Head and neck cancers (HNC) constitute 5–50% of all cancers globally [1], and 5–8% of total body cancers in Europe and America [2-4]. In India, HNC constitutes about 30% of all cancers [5]. In some part of Northern Nigeria, a yearly hospital incidence of 20–24 new cases has been reported [6,7] while in southern part of the country HNC form 6.2% of all cancers [8]. In South-western Nigeria a yearly incidence of 33–38 new cases has been reported [9,10]. Nigeria is a developing country in Africa and its people are of the Negroid race. Nigeria has a population of 140 million persons and a land area of 923,768.64 sq. kilometers [11]. There are six geopolitical zones in the country and all the states in the country have primary, secondary and tertiary health care institutions. Major occupations in Nigeria include agricultural, administrative jobs, manufacturing, white-collar professional jobs and various artisan jobs. Mining which is limited to few areas of the country is poorly developed [11].

Occurrence of cancers at various sites in the body differs from one place to another even in the same country [6,7,9,10]. Aetiological factors associated with cancers vary according to major risk factors in different geographical areas and between genders [7,9,12,13]. As demographic factors change, the pattern of cancers in a place may also change.

It is difficult to appreciate the problem of cancers in Nigeria because there is lack of comprehensive data and most studies available are hospital-based studies. There are no community based or population based database, previous studies were region-based and not nation-based and most of these reports are retrospective. A clearer understanding of the patterns of cancers will assist providers of health care as they plan the management of cancers. Such epidemiological data and other information can also be used to guide the future funding of public health programmes geared towards prevention and management of cancers.

The aim of this study is to highlight current knowledge on the burden of head and neck cancers in Nigeria and to discuss potential health care actions to improve prevention and treatment.

## Methods

A literature search using Medline was conducted for publications on head and neck cancers in Nigeria. The relevant references in the identified publications were manually searched for other additional relevant non-Medline articles or abstracts. Personal contacts were also made with authors of previous studies for the provision of articles found suitable for the review. The full-texts of all of these articles were thoroughly examined by the authors for the occurrence, distribution, identified risks factors, presentations, diagnostic method, treatment, prognosis and challenges associated with the management of HNC.

## Results

A total of 27 relevant published articles on head and neck cancers from 1968 to 2008 were reviewed. Ten of them were on the pattern of head and neck cancers from different regions [6-10,12,14-18] (Additional file 1), while others were on specific sites namely nasopharynx, sino-nasal, larynx, oral cavity and salivary glands from different regions[13,19-34] (Additional file 2).

### Epidemiology

A review of the published literature from Nigeria showed that the age of patients ranged between nine months to over 80 years [6-10,12,13,19-35]. It is known generally that cancers are diseases of the elderly. But reports from Jos and Maiduguri in Northern Nigeria [6,8,14] show a peak incidence in the 3^rd ^and 4^th ^decades, those from Lagos (South west) and Ilorin (North central) [10,16] reported a peak incidence in the 4^th ^to 5^th ^decades while reports from Ibadan (South west) Nigeria [12,18] showed a peak incidence in the 6^th ^decade of life. The peak age of incidence of Nasopharyngeal malignancies in most reports from Nigeria was found in the 4^th ^decade of life [9,10,15,21-23]. However, a bimodal curve was reported from Jos (North central) and Lagos (South west) with the first peak in the 2^nd ^decade and the second peak in the 4^th ^and 5^th ^decade [21-23]. Laryngeal cancers had a peak incidence in the 5^th ^decade in South-western cities of Lagos and Ibadan [10,28], while in northern city of Jos [26] the mean age was 46.5 years with peak incidence in the 4^th ^decade. The peak age occurrence of maxillary cancer was in the 4^th ^decade in Jos [24] and in the 5^th ^decade and the 6^th ^to 7^th ^decades in two different reports from Lagos [10,25]. A recent study from Lagos showed that the peak age of incidence of oral cancer in Lagos was in the 5^th ^decade of life [29]. In this study the age of patients ranged from 2.5 to 85 years, and 25% of the cases were found in patients below the age of 40 years [29]. A review of squamous cell carcinoma of the oral cavity in Lagos found the peak incidence in the 20 to 29-year and 40 to 49-year age groups, with 40% cases occurring in patients under age of 40 years [30]. The mean age of occurrence of salivary gland tumours in Enugu and in Lagos was 40 years [32-34].

In most studies from Nigeria, HNC affected more males than females [6,8,10,12,14,15,17,35,36], except a study from Ilorin (North central) [16], where females were affected more than males. The male to female ratio ranged from 1:1 to 2.3:1 [6,8,10,12,14-16,29,30,36].

### Aetiological Factors

Identified risks factors among the reviewed articles included: kola nuts, tobacco, farming, viral infections, alcohol and smoking [7,9,12,13,23,26,35]. In a report from Maiduguri [7] northern region of the country, tobacco smoking, tobacco chewing and chewing of kola nuts were associated with carcinoma of oral cavity. Kola nuts (Cola acuminate) has been reported to promote palatal mucosa keratinization of cigarette smokers and is considered a co-carcinogen. Laryngeal cancer was commoner in patients who consumed alcohol than smokers in the report from Jos [26], while in reports from Enugu [35], Lagos [13] and Ile Ife [9] most patients with laryngeal carcinoma were non-smokers. Virus is thought to be responsible for some HNC in sero-positive patients by viral oncogenesis. Nwaorgu et al [12] reported that salivary gland malignancy was the commonest tumour in patients with HIV sero-positivity in Ibadan (South-west) while Otoh et al [7] reported Kaposi sarcoma as the most common tumours in these patients in North-eastern Nigeria.

### Tumour Sites

Reports on the overall pattern of Head and neck cancers from different regions of the country cited nasopharynx as the commonest site [7,8,10,12,14,15] (Additional file 3). The nose and paranasal sinuses were the second most common reported sites [7,10,12,14] while larynx, was the third commonly affected site [8,10,14,17]. In contrast, Amusa et al [9] and Otoh et al [7] reported differently that malignancy of the oral cavity was the commonest in Ile-Ife (South-west) and Maiduguri (North central) Nigeria. Ologe et al [16] and Otoh et al [7] reported that thyroid is not an uncommon site of HNC. A review on commonest sites of oro-facial malignancy from Lagos, noted the mandible, maxilla, palate, tongue, cheek, lip and floor of the mouth [29,30] as the most commonly affected sites. Tumours of the oropharynx, hypopharynx, skin and eye were also seen but in low in prevalence in some reports [7,8,10,12,14-17]. A recent study [18] from Ibadan (South-west) reported that oral cavity and oropharyx were the most commonly affected sites accounting for 31.1% of cases, followed by nasopharynx (16.4%) and nose/paranasal sinuses (15%). Cancers of the ear was notably few [7-9,14,17]. Unknown metastatic neck nodes was reported in some of the series [7,9,10,12,15,16].

### Tumour Type

The majority of HNC was epithelial in origin and was mostly squamous cell carcinoma (SCC) [7-10,14-16,18]. SCC constituted 66.7% of all epithelial tumours in a recent study from Ibadan (South-west), Nigeria. Lymphomas were the second most frequent cell type seen in many centres [6,7,9,14,15]. Nwawolo et al in Lagos, Lilly-Tariah in Jos and Okoye et al in Port-harcourt found sarcomas to be the second most occurring histologic type [8,10,17]. The two most common head and neck cancers in Ibadan were SCC, which accounted for 47.8% of all cases, followed by lymphomas which accounted for 19.3% [18]. By contrast, Amusa et al [9] found that lymphoma was the most frequently diagnosed head and neck cancer, accounting for 40.3%, whereas SCC only accounted for 25% of cases. Ajayi et al [29] from Lagos reported epithelial malignant tumours (69%), mostly SCC as the most common orofacial malignant tumours followed by sarcomas (18%) and lymphomas (13%).

### Presentation, Diagnosing and Staging

Few studies reported on the clinical presentations and staging of head and neck tumours [7,13,23,25,26]. Late presentation of the advanced disease was a common feature in most reports from different parts of Nigeria. [7,13,19,20,22,23,25,26]. By the TNM classification of tumours, only 1 study reported stage 1 tumours among all the studied reports [13]. Stage II cancers were few and the reported sites were larynx and nasopharynx [7,13,22,27]. Most reports were on stages III and IV and the reported sites were larynx, nasopharynx and maxillary antrum [7,13,19,20,25,27].

### Treatment and Outcome

There are few reports on the treatment and outcome of HNC in Nigeria [7,13,15,19,20,22,23,25,27]. Otoh et al [7] in North central Nigeria reported that radiotherapy, surgery and chemotherapy were the main therapeutic modalities for carcinoma, sarcoma and lymphoma respectively. In their study, radiotherapy was used in 41.7%, surgery 39.6%, combination of radiotherapy/surgery in 11.4% and chemotherapy in 7.3% [7], however, treatment outcome was not reported. Iseh and Malami [15] reported that surgery and chemotherapy were the main treatment modalities in Sokoto (North-west, Nigeria), and adduced non availability of radiotherapy in the region and the logistics of assessing the radiotherapy facility as the reasons for the treatment modalities in the report. Report on laryngeal carcinoma treatment in South western Nigeria [13] showed 86.1% of patients had radiotherapy, 2.7% had laryngectomy and 8.3% received a combination of surgery and radiotherapy. Of these cases [13] 47.3% of the patients were lost to follow up less than two years, 16.7% after two years, 22.2% were dead after two years of treatment of which 5.6% were unrelated to the carcinoma. Recurrence rate after 2 years was 16.7%. Alive and disease free 4 years post-treatment was 5.6%. Treatment modality for laryngeal carcinoma from North central Nigeria [27], showed that 31.5% had total laryngectomy, 20.4% were inoperable and were referred for radiotherapy and 48% refused surgery. Almost all the patients were lost to follow up after 1 year [27]. Radiotherapy is the mainstay of nasopharyngeal carcinoma [21] except where such is not available [15,19,20,23]. Lilly-Tariah et al [24] on report on treatment of naso-antral carcinoma from north western Nigeria: 33.3% had surgery (hemimaxillectomy/frontoethmoidectomy), 41.7% had chemotherapy alone, and 5.6% received a combination of radiotherapy and chemotherapy. Management of maxillary antral carcinomas as reported by Ogunlewe et al [25] in Lagos (South west, Nigeria) included hemi-maxillectomy and radiotherapy in 68.2% of cases, hemi-maxillectomy/orbital exenteration and radiotherapy in 10.53% of patients, and radiotherapy and chemotherapy in 13.6% of cases. After 2 years, recurrence rate was 5.3%, death due to the disease occurred in 10.5% of cases and 84.2% was lost to follow up during the period.

## Discussion

### Historical perspectives

One of the earliest references to any head and neck cancers in Nigeria was the work of Elmes and Baldwin [36] who reported cases of nasopharyngeal cancer but noted that it was rare. Edington and Gilles (1965), and Edington and Maclean (1965) also reported cases of oesophageal carcinoma and salivary gland tumours respectively and found them to be rare in Nigeria [31,37]. The work of Martinson in Ibadan in 1968 showed that cancer of the nasopharynx was not so uncommon as previously reported [19]. The pioneering work of Okafor in South-eastern Nigeria pointed to malignant tumours being rare in the HNC [35]. He did however, noted that all the cases presented late to the hospital [35].

University College Hospital (UCH) Ibadan was the first teaching hospital in Nigeria, and is also the first hospital to be equipped with radiotherapy unit and Computed Tomography-scan facility among other facilities. The effect of this is that all across the country, patients were referred to Ibadan for diagnosis and irradiation therapy for a long period of time. The first cancer registry was also established in 1960 in UCH, Ibadan [20]. The diagnosis and management of HNC rests on the head and neck surgeons and the pathologists. There are currently less than 200 Ear, Nose and Throat Head/Neck surgeons, less than 250 pathologists and less than 30 radiation oncologist and clinical therapist practicing in the country. In addition, there are fewer than 50 practicing Oral and Maxillofacial Surgeons in the country. All these are insufficient for a population of over 140 million people.

It is difficult to appreciate the problem of cancers in Nigeria because most studies available are hospital-based studies [21]. There are no community based or population based database. Most of the reports are retrospective and a lot of the data might have been lost.

### Epidemiology

Overall, Head and Neck Cancers seem to affect Nigerians at a younger age than in Caucasians. This is in keeping with reports from other African studies [38,39]. The reasons for higher percentage of occurrence in younger age group in Nigeria are not so clear but they may be connected to issues of race, genetics, poverty and behavioral practices. Shorter life expectancy in Africans compared with Caucasians, and earlier exposure to risk factors have also been speculated [29]. However, it has also been reported that oral squamous cell carcinoma (SCC) is now on the increase in younger age groups in United Kingdom and USA [40].

There is male preponderance in most studies of HNC worldwide [41-43] in agreement with reports from Nigeria. The reason for this is not obvious. In Nigeria, males and females are exposed to the same factors. There are preferences in activities but these are not exclusive. Men consume more alcohol and tobacco than females. It is possible that hormonal factors may play a significant role.

### Aetiological Factors

The role of alcohol and tobacco in carcinogenesis is well documented [4,44,45]. Nigerians smoke cigarettes but not as much as in the western world. In all the studies, there was no proven direct relationship with smoking. Alcohol is more indigenous and its usage is more widespread. Carcinoma of the larynx when it occurs in non-smokers has been found to be biologically aggressive [13]. The role of the Epstein Barr virus and human papilloma virus in HNC are well documented in the literature [44,45], but no such study has been reported in Nigeria. The role of hard wood dust in adenocarcinoma of the nose and paranasal sinuses, nickel and chromate dust in the larynx, nose and paranasal sinuses and hydrocarbons in HNC are well known [4,44,45] but these were not determined in all the studies so far. As Nigeria continues to industrialize, the role of these agents in carcinogenesis cannot be ignored.

### Tumour sites

A diversity of cancers of anatomical sites is included under the broad group "head and neck cancer" by various authors. For example, Onyango et al [41] included eye and thyroid cancers. Nwawolo et al [10] included neither cervical esophageal, thyroid nor eye malignancies in this group of lesions. Adeyemi et al [18] did not separate oral cancers from oropharyngeal cancers in their reports. However, most reports across the country confirms nasopharynx, sinus-nasal and larynx, as the commonly affected sites of HNC in Nigeria [10,12,14-17] while the reported pattern in Kenya was Larynx, oral cavity and nasopharynx [43].

### Tumour Type

SCC was the most prevalent histologic type in most of the reports from Nigeria [10,12,14-18] This is in agreement with the literature worldwide [41-45]. However, 2 studies reports from Nigeria have reported lymphomas as the most common HNC cancers followed by squamous cell carcinoma [6,9].

### Regional Differences

The population and land size of Nigeria is large and the customs and practices are just as varied. The climatic conditions from one place to the other also vary. In the rural areas the people are largely indigenous to the area. In the urban centres this is not quite the case as this is a melting point of all Nigerians. There are tertiary/teaching hospitals in all the geo-political zones of the country and head and neck surgeons are available in all of them. These hospitals are in the urban and semi-urban areas, and funding and provision of diagnostics facilities is the prerogative of the federal government. The reports from various centres across the country as reviewed in the present study are hardly a true reflection of the cancer pattern of any particular tribe or groups. The nature of the studies such as the study design (mostly retrospective in nature), small sample size and short duration of study are some of the factors that make it difficult to make out any clear-cut disease pattern. There were more similarities than differences in the pattern of HNC across the country. The observed minor differences could hardly be said to be due to geographical difference. In a report from Ibadan [18], nasopharyngeal cancers were the most common HNC. In contrast, studies from Ilorin and Ile-Ife reported nasal/paranasal cancers and oral cancers as the most frequently occurring HNC respectively. In addition, in the same center, patterns may vary at different times; the first study in Jos [6] identified neck lymphomas as the most common HNC, whereas a later work [8] showed nasopharyngeal cancer to be the commonest. The import of this is that there is a need for a large scale national survey on the incidence, aetiology, pattern of presentation, management and prognosis of Head and Neck Cancers. These research questions are best answered by either a longitudinal, cohort or a case-control study [46].

### Diagnosing and Staging

Cancers are known to occur more often with increasing age [40,45]. The presenting features will depend on the primary site of the disease. Sometimes the lesion is so gross that it is difficult to determine the primary source. One common feature of HNC is unknown primary lesion. The early symptoms of HNC are usually vague and non-specific. Because these symptoms do not produce functional limitation or cosmetic problems, they are often ignored or not suspected. A lot of primary physicians are not familiar with these lesions. In this part of the world, patients consulting traditional healers and spiritual faith healers tend to contribute to needless delays.

Sadly, most patients in our environment present at advanced stage of the [7,13,31]. Ignorance, poverty and lack of trained personnel (ENT surgeons, maxillofacial surgeons, general surgeons, and family physicians) to make proper diagnosis and referral contribute to the dismal state of late presentation. The diagnosis of HNC requires a good knowledge of the disease pattern and a high index of suspicion. Some sites of HNC like larynx, pharynx and maxillary antrum are not readily visible or palpable requiring examination under anaesthesia. Video-endoscopic examination of mucosal surfaces, imaging technique, multiple biopsies of suspected primary sites and histological diagnosis are some of the requirements for diagnosis. The skill and material for these are not readily available in most of the hospitals of first contact. CT scan and MRI are revolutionary tools for radiologic diagnosis and determination of the extent of treatment and the progress of the disease. A total of about thirty CT scanner machines and about fifteen MRI machines are available across the country, and about 70% of these are located in the southern part of the country. Till date, considerable distances, sometimes over 500 kilometres, have to be covered to get to a center that has a functioning CT scan and MRI facilities.

The advent of immunohistochemistry and immunocytochemistry has also revolutionized the histological diagnosis of head and neck Cancers. Differentiation of poorly differentiated or anaplastic carcinoma from histological subtypes of sarcomas and lymphomas may require immunohistochemical staining [47]. However, facilities for this special staining technique are not readily available in most centres across the country [7].

### Treatment

Surgery, radiotherapy and chemotherapy either alone or in combination are the standard modalities of treatment of HNC [48,49]. The histologic cell type and the stage of the disease determine the choice of treatment. Lymphomas respond satisfactorily to chemotherapy. Early stages of head and neck carcinomas can be treated satisfactorily (cured) with radiotherapy alone or surgery alone. Stages III and IV carcinomas require a combination of salvage surgery and radiotherapy [48]. Radiotherapy maybe used before or after surgery [45,49]. Chemotherapy is also useful in cases of metastasis and recurrence. Chemotherapy may also be used for induction chemotherapy before radiotherapy [48]. Frozen section is a standard procedure to ascertain safety or otherwise of tumour margins in most centres around the world. In Nigeria, frozen sections are not routinely done in the management of HNC.

### Outcome of Treatment

There are few reports on the outcome of HNC treatment in Nigeria. Most patients in this environment present at hospitals at a late stage of the disease [22,27]. Treatment at this stage is largely palliative and not curative. This certainly carries a poor prognosis. High drop-out rate is another problem in this environment. The reasons include poverty, ignorance, and difficulty in getting the appropriate attention from the few personnel available, long distances to the health centers and the high cost of drugs when they are available. Treatment of cancers in Nigeria was free about 2 decades ago but that has changed and the patients most of whom are poor have to pay for these expensive and scarce drugs [21]. Complications from treatment following surgery, radiotherapy or chemotherapy are many and can be severe. Over dosage (>50–59 Grays) maybe a cause of morbidity and mortality [22], while under-dosage may be a cause for early recurrence and mortality.

### Other Medical Conditions

Chronic liver, lung and heart diseases may affect management of HNC. In addition, chronic alcohol usage maybe a factor in the management and aetiology of HNC [45]. Diabetes mellitus, hypertension and HIV/AIDS are endemic conditions that affect the management of HNC. These conditions call for a multidisciplinary approach.

### Funding the Burden

The burden of managing HNC is enormous. Annually, more than 10 million persons are diagnosed with cancer [21]. More than half of these persons are in the developing world [21]. The World Health Organization (WHO) estimates that 12.5% of all deaths worldwide are due to cancer. This is greater than HIV/AIDS, malaria and tuberculosis combined [21,46,50]. The disease is on the increase and the peak incidence involves the active working class. The diagnosis of HNC is capital intensive. Only the Government, the WHO, International Atomic Energy Agency (IAEA) and such organizations can bear this cost. This involves planning, quality control programme, training of manpower and putting in place the necessary infrastructures. In a situation where only 5% of cytotoxic drugs get to the developing world, which has 50% of all cancers, only governments can intervene. There are five functioning radiotherapy centers in Nigeria today with a population of over 140 million of which three are located in the south west, one in the middle belt and one in the north. This comes to one radiotherapy unit to 30 million persons as against the WHO recommendation of 1 to 250,000 persons [21]. It has only recently been estimated that it costs about $100 billion a year to treat patients with cancers overseas [51].

### Prospects for the Future

Head and neck cancers are not uncommon in Nigeria. Reports from centers across the country showed that cancers of the nasopharynx, sinonasal and larynx are the most frequently seen. Most patients present late for diagnosis and treatment; and subsequently prognosis of HNC in our environment is poor. The WHO reported that a third of all cancers are preventable, and therefore prevention programmes target at reducing the incidence of cancers should be given a priority by the appropriate authority. There must be planning and the political will to enforce the plans. There must be an effective National Cancer Registry. Without the statistics on HNC, it will be impossible to plan meaningfully. The government should set up the National Cancer Institute with a view to educating the public on cancer prevention, detection and treatment. There should be a policy on training more head and neck surgeons, pathologists, radiation oncologist and clinical therapist and other support staff. The challenges of HNC are enormous and the governments at various levels must take active part in intensive public enlightenment campaign aimed at reducing the incidence and burden of head and neck cancers. Finally, it would be of great benefit if a population-based study were undertaken to determine the national incidence of head and neck cancer in Nigeria. Most of the data available in the literature are based on hospital studies that are difficult to relate to the general population. Longitudinal and cohort studies are also needed to identify possible risk factors for the development of cancer of the head and neck region in our population.

## Competing interests

The authors declare that they have no competing interests.

## Authors' contributions

OB conceived of the study and participated in the design and coordination. AO and WL participated in the design of the study and in the search for the literature used for the writing of the manuscript. WL and AO coordinated the online submission of the manuscript. All authors read and approved the final manuscript.

## Supplementary Material

Additional file 1 **Publications on pattern of head and neck cancers in geopolitical region in Nigeria**. The table shows publications on pattern of head and neck in Nigeria. Click here for file

Additional file 2 **Publications on specific sites of head and neck cancers in Nigeria**. The table shows publications on specific sites of head and neck cancers in Nigeria. Click here for file

Additional file 3 **Prevalence of different histologic subtypes of Head and Neck cancers across the geopolitical zones**. The table shows the prevalence of different histologic subtypes of head and neck cancers across the geopolitical zones of Nigeria. Click here for file
