# Supplementary material for: Current evidence on the burden of head and neck cancers in Nigeria
Source: Head Neck Oncol. 2009 May 28;1:14. doi: 10.1186/1758-3284-1-14 (PMC2694192; doi:10.1186/1758-3284-1-14)
Supplement: Additional file 1 — Publications on pattern of head and neck cancers in geopolitical region in Nigeria. The table shows publications on pattern of head and neck in Nigeria. [file 1758-3284-1-14-S1.doc]

**Table 1: Publications on pattern of head and neck cancers in geopolitical region in Nigeria**

Author Year of Region of No of Duration of 3 Commonest reported

Publication study Patient study (years) sites of cancer……….

Bhatia [6] 1990 NCN 73 3 1. Neck Lymphoma

2. Sinonasal

3. Salivary

Otoh [7] 2004 NEN 317 5 1. Oral cavity

2. Nasopharynx

3. Sinonasal

Lilly-Tariah [8] 1999 NCN 36 10 1. Sinonasal

2. Nasopharynx

3. Larynx

Amusa [9] 2004 SWN 313 10 1. Oral cavity

2. Neck Lymphoma

3. Thyroid

Nwawolo [10] 2001 SWN 381 10 1. Nasopharynx

2. Sinonasal

3. Larynx

Nwaorgu [12] 2007 SWN 521 10 1. Nasopharyx

2. Larynx

3. Sinonasal

Ahmad [14] 2004 NEN 77 12 1. Nasopharynx

2. Sinonasal 3. Larynx

Iseh [15] 2006 NWN 131 5 1. Nasopharynx

2. Neck Lymphoma

3. Oral cavity

Ologe [16] 2005 NCN 89 5 1. Sinonasal

2. Thyroid

3. Nasopharynx

Okoye [17] 1995 SSN 21 2 1. Sinonasal

2. Nasopharynx 3. Larynx

Adeyemi [18] 2008 SWN 972 3 1. Oral/oropharyngeal

2. Nasopharyngeal

3. Sinonasal

**Keys** NCN=North central Nigeria, NWN=North western Nigeria, NEN=North eastern Nigeria

SSN= South south Nigeria, SWN=South western Nigeria, SEN=South eastern Nigeria
