# Supplementary material for: Current evidence on the burden of head and neck cancers in Nigeria
Source: Head Neck Oncol. 2009 May 28;1:14. doi: 10.1186/1758-3284-1-14 (PMC2694192; doi:10.1186/1758-3284-1-14)
Supplement: Additional file 2 — Publications on specific sites of head and neck cancers in Nigeria. The table shows publications on specific sites of head and neck cancers in Nigeria. [file 1758-3284-1-14-S2.doc]

**Table 2: Publications on specific sites of head and neck cancers in Nigeria**

Author Year of Region of the No of patient Duration of Reported site of cancer

publication Country studied study (yrs)

Somefun [13] 2003 SWN 36 4 Larynx

Martinson [19] 1968 SWN 55 5 Nasopharynx

Martinson [20] 1984 SWN 180 15 Nasopharynx

Okeowo [21] 1978 SWN 48 10 Nasopharynx

Ketiku [22] 1993 SWN 98 10 Nasopharynx

Lilly-Tariah [23] 2003 NCN 55 10 Nasopharyx

Lilly-Tariah [24] 1999 NCN 36 10 Sinonasal

Ogunlewe [25] 2001 SWN 38 5 Sinonasal

Lilly-Tariah [26] 1999 NCN 54 12 Larynx

Lilly-Tariah [27] 2002 NCN 38 13 Larynx

Nworgu [28] 2002 SWN 72 10 Larynx

Ajayi [29] 2007 SWN 256 10 Oral cavity

Effiom [30] 2008 SWN 233 10 Oral cavity

Sowemimo [31] 1978 SWN 38 14 Salivary gland

Ezeanolue [32] 1999 SEN 41 10 Salivary gland

Somefun [33] 2007 SWN 58 12 Salivary Parotid gland

Ladeinde [34] 2007 SWN 120 15 Salivary gland

**Keys** NCN=North central Nigeria, NWN=North western Nigeria, NEN=North eastern Nigeria

SSN= South south Nigeria, SWN=South western Nigeria, SEN=South eastern Nigeria
