# Supplementary material for: Current evidence on the burden of head and neck cancers in Nigeria
Source: Head Neck Oncol. 2009 May 28;1:14. doi: 10.1186/1758-3284-1-14 (PMC2694192; doi:10.1186/1758-3284-1-14)
Supplement: Additional file 3 — Prevalence of different histologic subtypes of Head and Neck cancers across the geopolitical zones. The table shows the prevalence of different histologic subtypes of head and neck cancers across the geopolitical zones of Nigeria. [file 1758-3284-1-14-S3.doc]

Table 3: Prevalence of different histologic subtypes of Head and Neck cancers across the geopolitical zones

| Author |  | Bhatia [6] | Otoh [7] | Lilly-Tariah [8] | Amusa [9] | Nwawolo [10] | Nworgu [12] | Ahmad [14] | Iseh [15] | Ologe [16] | Okoye [17] |
| --- | --- | --- | --- | --- | --- | --- | --- | --- | --- | --- | --- |
| Region of the Country |  | NCN | NEN | NCN | SWN | SWN | SWN | NEN | NWN | NCN | SSN |
| Sites | **ICD – 10No** | No. % | No. ( %) | No. ( %) | No. (%) | No. (%) | No. ( %) | No. ( %) | No. ( %) | No. ( %) | No. % |
| Oral cavity | C00-66 | 4 (5.5) | **48 (15.1)** | 8. (7.8) | **115 (33.8)** | 36 (6.5) | 8 (1.5) |  | **9 (6.9)** | 5.5 (6) |  |
| Nasopharynx | C 11 | 8 (10.9) | **34 (10.7)** | **20 (19.6)** | 10 (3.2) | **64 (16.8)** | **115 (22.1)** | **36 (46.8)** | **15 (11.5)** | **10 (11.3)** | ***4 (19.1)*** |
| Hodgkins  Non Hodgkins | C81  C82 C83 C85 | **23 (31.5)** | 33 (10.4)  21 (6.6) |  | ***50 (15.9)*** | 9 (2.4) | 16 (3.1) |  | ***9 (6.9)*** |  |  |
|  |  |  | 54 (17) |  |  |  |  |  |  |  |  |
|  |  |  |  |  |  |  |  |  | 7 (5.3) |  |  |
| Eye & Adenexa | C69 |  | 30 (9.5) |  | 15 (4.8) | 13 (3.4) |  |  | 7 (5.3) | 2 (2.2) |  |
| Maxillary sinus/Nose | C3D & C31 | ***18 (24.7)*** | ***40 (12.6)*** | ***23 (22.5)*** | 1 (0.3) | ***52 (13.6)*** | **91 (17.5)** | ***28 (36.4)*** | 7 (5.3) | **16 (18)** | **9 (42.9)** |
| Jaw bones | C41 |  | 21 (6.6) |  |  | 28 (7.3) |  |  | 8 (6.1) |  |  |
| Salivary glands | C07 C088 | 9 (12.3) | 18 (5.7) | 10 (9.8) | 5 (1.6) | 29 (7.6) | 11 (2.1) |  | 4 (3.1) | 1 (1.1) |  |
| Thyroid | C73 |  | 17 (5.4) |  | **43 (13.7)** | 28 (7.3) | 15 (2.9) |  | 4 (3.8) | ***11 (12.4)*** |  |
| Larynx | C32 | 4 (5.5) | 15 (4.7) | **17 (16.6)** | 6 (1.9) | **46 (12.1)** | ***163 (31.3****)* | **7 (9.1)** | 2 (1.5) | 4 (4.5) | **2 (9.5)** |
| Oropharyx | C10 | 4 (5.5) | 5 (1.3) | 4 (5.2) |  | 14 (3.7) | 35 (6.7) | 4 (5.2) |  | 1 (1.1) | 1 (4.8) |
| Hypopharynx | C13 | 2 (2.7) | 3 (0.9) |  |  | 1 (0.3) | 19 (3.7) |  |  | 3 (3.4) | 2 (9.5) |
| Other lymphomas | C84 |  | 2 (0.6) |  |  |  |  |  |  |  |  |
| Leukemia | C95 |  | 1 (0.3) |  |  |  |  |  |  |  |  |
| Tonsil | C09 |  | 1 (0.3) |  | 10 (3.2) |  |  |  |  |  |  |
| Eye lid |  |  |  |  |  |  |  |  |  |  |  |
| Skin | C44 |  | 7.0 |  | 7 (2.2) | 35 (9.2%) |  |  |  | 1 (1.1) |  |
| Ear |  |  | 1 | 2 (2.7) | 1 (0.3) |  | 9 (1.7) | 2 (2.7) |  |  | 1 (4.8) |
| Unknown Metastatic nodes |  |  | 8 (2.5) |  | 15 (4.6) | 11 (2.9%) | 25 (4.8) |  | 3 (2.3) | 9 (10.1) |  |

**Keys** NCN=North central Nigeria, NWN=North western Nigeria, NEN=North eastern Nigeria

SSN= South south Nigeria, SWN=South western Nigeria, SEN=South eastern Nigeria

Bold= commonest HNC,

Bold Italics = second commonest HNC

Bold underline =third commonest HNC
